# The Cytokine Flt3-Ligand in Normal and Malignant Hematopoiesis

**DOI:** 10.3390/ijms18061115

**Published:** 2017-05-24

**Authors:** Panagiotis Tsapogas, Ciaran James Mooney, Geoffrey Brown, Antonius Rolink

**Affiliations:** 1 Developmental and Molecular Immunology, Department of Biomedicine, University of Basel, Mattenstrasse 28, Basel 4058, Switzerland; antonius.rolink@unibas.ch; 2 Institute of Immunology and Immunotherapy, College of Medical and Dental Sciences, University of Birmingham, Edbgaston, Birmingham B15 2TT, UK; c.mooney@smd15.qmul.ac.uk (C.J.M.); g.brown@bham.ac.uk (G.B.); 3 Institute of Clinical Sciences, College of Medical and Dental Sciences, University of Birmingham, Edbgaston, Birmingham B15 2TT, UK

**Keywords:** Flt3, hematopoiesis, acute myeloid leukemia (AML), cytokines, Flt3 with internal tandem duplications (FLT3-ITD)

## Abstract

The cytokine Fms-like tyrosine kinase 3 ligand (FL) is an important regulator of hematopoiesis. Its receptor, Flt3, is expressed on myeloid, lymphoid and dendritic cell progenitors and is considered an important growth and differentiation factor for several hematopoietic lineages. Activating mutations of Flt3 are frequently found in acute myeloid leukemia (AML) patients and associated with a poor clinical prognosis. In the present review we provide an overview of our current knowledge on the role of FL in the generation of blood cell lineages. We examine recent studies on Flt3 expression by hematopoietic stem cells and its potential instructive action at early stages of hematopoiesis. In addition, we review current findings on the role of mutated FLT3 in leukemia and the development of FLT3 inhibitors for therapeutic use to treat AML. The importance of mouse models in elucidating the role of Flt3-ligand in normal and malignant hematopoiesis is discussed.

## 1. Introduction

Leukemias occur as a result of the de-regulation of normal hematopoiesis, as evidenced by the significant number of genes important for hematopoietic development that are mutated in leukemias. Therefore, understanding how hematopoiesis is regulated is of utmost importance for the elucidation of the mechanisms that lead to the blood cell malignancies. Cytokines are important regulators of hematopoietic development: they transfer extra-cellular signals to cells to affect their survival, proliferation, differentiation and maturation [1]. The cytokine “Fms-like tyrosine kinase 3 ligand” (hereafter FL) represents a typical example of a hematopoietic cytokine whose receptor is often found to be mutated or over-expressed in leukemias. Therefore, there has been a great deal of attention on the precise role of FL in hematopoiesis.

In this review we will give a general outline of our current knowledge of the role of FL in normal and malignant hematopoiesis and will briefly discuss recent findings from us and others that offer new insights on how FL regulates the generation of blood cells. Furthermore, the potential relevance of these findings to the role of FL in hematopoietic malignancies will be discussed.

## 2. FL and Its Receptor, Flt3

To date, only one receptor for the cytokine FL has been identified; Fms-like tyrosine kinase 3 (also known as Flk2/CD135; hereafter Flt3). The murine receptor was the first to be identified and cloned by two groups independently [2,3,4], and soon after its human homologue was cloned [5]. Flt3 belongs to the class III receptor tyrosine kinase group of receptors, which also contains other cytokine receptors, such as the platelet-derived growth factor receptor (PDGF) and c-kit, the receptor for Stem Cell Factor (SCF). Flt3 shows a high sequence and structural homology to these receptors, containing an extra-cellular part with 5 immunoglobulin-like domains, a transmembrane domain and a cytoplasmic region consisting of a juxta-membrane domain followed by two tyrosine kinase domains [6]. The pattern of expression of Flt3 observed in early studies was indicative of its potential importance in hematopoiesis, as it was found to be expressed specifically by early hematopoietic progenitors that contain some degree of stem cell activity, while it was not expressed by more mature blood cell populations [2,3]. In 1993, murine FL was cloned and characterized as a transmembrane protein that can be secreted and, upon binding to Flt3, can stimulate the proliferation of Flt3^+^ bone marrow and foetal liver progenitors [7,8]. Soon after the human FL was cloned; interestingly, it was shown to be able to bind to murine Flt3 and activate its downstream signaling [9].

Binding of FL to Flt3 leads to homodimerization of the receptor and subsequent conformational changes that result in phosphorylation of the tyrosine kinase domains. This activation of the receptor occurs rapidly and is followed by an equally rapid internalization and degradation of the receptor homodimer [10]. Downstream signaling pathways that are activated by FL-Flt3 binding were studied even before FL was cloned, using a chimeric Flt3 receptor containing the extracellular part of Colony Stimulating Factor 1 receptor (CSF-1R) [11,12]. Subsequent experiments using the native receptor have confirmed some of the initial findings and revealed that Flt3 activation leads to phosphorylation of Src-homology 2 containing proteins (SHC), which directly interact with proteins such as Grb2, Gab2, SHIP, eventually leading to activation of the Ras/MEK/Erk and PI3K pathways [13,14,15,16]. Human FLT3 in particular has been shown to bind additionally to tyrosine phosphatase SHP2 and the proto-oncogene CBL, also members of the PI3K pathway [15,17,18]. In addition, Flt3 has been shown to activate Stat3 [19,20] and Stat5a [21] signaling mediators. However, caution should be exercised when interpreting these findings on Flt3 signaling, as most of the above data are derived from work using cell lines, while additional complexity comes from the fact that downstream signaling pathways activated by Flt3 seem to be highly cell-context dependent.

The cloning of FL allowed the investigation of its function and early studies focused mainly on assessing its role in supporting the growth and differentiation of hematopoietic progenitors in vitro. Several of these studies demonstrated that FL has a limited effect on the growth of hematopoietic progenitors when used alone, but it is very potent in synergizing with other hematopoietic cytokines, such as SCF, IL-3, IL-6, Granulocyte-Macrophage Colony stimulating Factor (GM-CSF), Granulocyte Colony Stimulating Factor (G-CSF) and IL-11, to promote the generation of primarily myeloid cell containing colonies [22,23,24,25,26,27,28]. There was no significant effect of FL as to promoting the in vitro generation of cells of the erythroid [25] or megakaryocyte lineages [22]. In addition, FL was shown to significantly enhance the in vitro generation of B cells, again mainly in combination with the cytokines SCF and IL-7 [26,29,30,31]. A similar picture of the in vitro effect of FL emerged from studies using human hematopoietic progenitors [23,32,33]. The synergistic effect of FL with other hematopoietic cytokines was a clear conclusion from these experiments. However, studies have been limited both by the lack of markers that could be used to isolate progenitors at different developmental stages and by the use of an exclusively in vitro approach to determine cell behavior.

Important insights on the role of FL came from the generation of knock-out mouse models. Initial analysis of mice with targeted deletion of the *Flt3* gene showed no significant perturbations in hematopoiesis, apart from a reduction in the numbers of early B cell progenitors and defective repopulation capacity of *Flt3*^−/−^ bone marrow cells upon transplantation into irradiated hosts [34]. In contrast, mice lacking FL showed a more pronounced phenotype, with a reduced overall generation of leukocytes, particularly decreased B cell progenitors, natural-killer cells (NK) and dendritic cells (DC) [35]. The apparent difference in the severity of phenotype between *Flt3^−/−^* and FL^−/−^ mice could be indicative of the existence of another receptor for FL or alternatively reflect differences between mouse strains. Subsequent detailed analysis of mice defective in Flt3 signaling showed that apart from committed B cell progenitors, FL is important for the generation and/or maintenance of their uncommitted precursors, CLP (Common Lymphoid Progenitors) [36] and EPLM (Early Progenitors with Lymphoid and Myeloid potential) [37], as well as of early multi-potent progenitors (MPP) within the Lineage^−^kit^+^Sca1^−^ (LSK) compartment [38,39]—all of these populations express Flt3 [40,41]. These in vivo studies have shown that active Flt3 signaling is not an absolute requirement for hematopoiesis to occur, but have nevertheless highlighted its importance in regard to several developmental steps in blood cell formation.

## 3. The Role of FL in Normal Hematopoiesis

### 3.1. Hematopoietic Stem Cells and Early Progenitors

**Figure 1:**
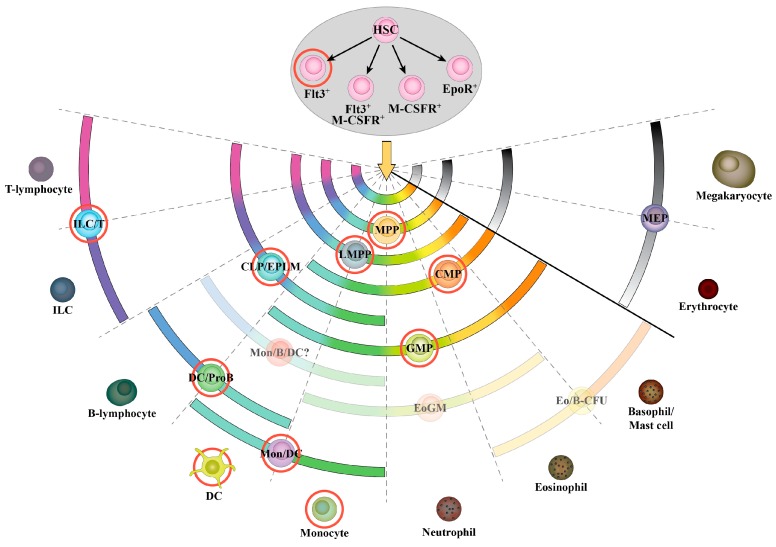
Flt3 expression in murine hematopoietic cells. Flt3 expression in progenitor and mature hematopoietic cells. The fate choices that are available to HSC are a continuum as shown by the short central arc below the yellow arrow. The fates choices of each of the indicated progenitors are shown as a shorter arc that spans the end cell types each progenitor cell population can give rise to. Red circles indicate Flt3 expression by the corresponding cell type. The grey section of the spectrum and grey shading of the MEP and mature cells indicates that these cells do not express Flt3. Progenitor cells that have not been investigated for expression of Flt3 are shown in a faded color. Expression is confined to myeloid and lymphoid progenitors as opposed to megakaryocyte/erythroid progenitors. HSC: Hematopoietic Stem Cell; MPP: Multi-Potent Progenitor; LMPP: Lymphoid-primed Multi-potent Progenitor; MEP: Megakaryocyte-Erythrocyte Progenitor; CMP: Common Myeloid Progenitor; GMP: Granulocyte-Macrophage Progenitor; CLP: Common Lymphoid Progenitor; EPLM: Early Progenitors with Lymphoid and Myeloid potential; ILC: Innate Lymphoid Cell; DC: Dendritic Cell; Eo: Eosinophil; CFU: Colony Forming Unit; Mon: Monocyte; M-CSFR: Macrophage–Colony Stimulating Factor Receptor; EpoR: Erythropoietin Receptor; GM: Granulocyte-Macrophage; ProB: progenitor B-lymphocyte; B: B-lymphocyte; T: T-lymphocyte.

The most broadly accepted model explaining how the generation of hematopoietic cells occurs from Hematopoietic Stem Cells (HSC) is based on a developmental hierarchy, with HSC residing at the apex as the multi-potent progenitor cell type that gives rise to all of the hematopoietic lineages through the step-wise generation of oligo-potent progenitors with restricted developmental potentials. This model is continuously debated and revised as new findings, often based on new technologies, provide new clues as to how hematopoiesis is regulated. Figure 1 illustrates Flt3 expression by different hematopoietic progenitors and lineages, based on our current knowledge and in the context of a continuum of options and the “pairwise” model for hematopoiesis we have proposed [42,43]. Investigation of Flt3 expression in hematopoietic progenitor stages has greatly contributed in identifying successive developmental stages in the hematopoietic pathway. For example, expression of Flt3 within the HSC-containing LSK compartment has been associated with loss of self-renewal capacity, therefore suggesting that the Flt3^−^ fraction of LSK cells is enriched for long-term reconstituting HSC (LT-HSC) [44,45].

The traditional model for hematopoiesis, which is the one most commonly found in textbooks, suggests an early bifurcation in the hematopoietic tree, with progenitors differentiating towards either a lymphoid fate, eventually giving rise to B, T and Innate Lymphoid (ILC) cells, or towards a myeloid fate, which results in the generation of all myeloid cells, platelets and erythrocytes. This model was based on the identification of distinct progenitor types, the CLP and the CMP (Common Myeloid Progenitor), which showed the above developmental potentials, respectively [46,47]. In 2005, the Jacobsen group reported that MPP progenitors with high levels of Flt3 expression (named Lymphoid-primed Multipotent Progenitors, or LMPP) have lost their potential to generate megakaryocytes and erythrocytes while retaining a robust lymphoid and myeloid potential (shown in Figure 1), thereby suggesting that the earliest branching point in hematopoiesis occurs between the megakaryocyte/erythrocyte and lymphoid/myeloid lineages [48]. Whether Flt3^+^ MPP progenitors can indeed give rise to cells of the megakaryocyte and erythrocyte lineages has been debated for some time [49,50,51,52]. Lineage tracing experiments have shown that all hematopoietic lineages, including megakaryocyte/erythrocyte cells, are derived from progenitors that at some point expressed *Flt3* mRNA [49,53]. However, these results could be explained by significant expression of *Flt3* mRNA prior to expression of the Flt3 protein (or a low, therefore FACS-undetectable, protein expression) on the surface of progenitors that give rise to megakaryocytes and erythrocytes.

The above data raise the issue of potential Flt3 expression by HSC. We have addressed this question by examining *Flt3* mRNA and protein at the single-cell level within HSC [54] phenotypically defined using the CD150/CD48 staining strategy first reported by the Morrison group [55,56]. Our findings have revealed that a small fraction of both LT- and short-term (ST)-HSC express Flt3 (Figure 1). These results are in agreement with a currently emerging view of heterogeneity within HSC [57,58] and have implications for our understanding of hematopoietic malignancies and the heterogeneity of leukemias, as discussed below. Interestingly, our single-cell analysis finds co-expression of Flt3 and the receptor for macrophage-colony stimulating factor (M-CSFR) by some HSC but virtually no co-expression of Flt3 with the receptor for the erythrocyte-lineage promoting cytokine erythropoietin (EpoR). In light of recent suggestions that lineage commitment might occur at a much earlier developmental stage than previously thought [59], these data indicate that some level of lineage skewing occurs already within the HSC population, with some cells being “primed” to respond to lineage-instructing cytokines and differentiate towards the corresponding cell fates.

The observed heterogeneity in Flt3 expression amongst early hematopoietic progenitors and its clear association specifically with the lymphoid and myeloid pathways (Figure 1) raises the question of whether FL has a functional role in promoting the differentiation of a particular cohort of hematopoietic progenitors. As mentioned previously, knock-out mouse models for both FL and Flt3 have shown a reduction in the numbers of early lymphoid and myeloid progenitors. This could be due to either a proliferative and/or survival role of FL on Flt3^+^ progenitors or it could reflect a function of the cytokine as a differentiating factor, since hematopoietic cytokines can promote the generation of different lineages by acting either in a permissive way (selective expansion of receptor-positive lineages through promotion of their proliferation and/or survival), or in an instructive way (activation of a lineage-specific genetic program) [60,61].

**Figure 2:**
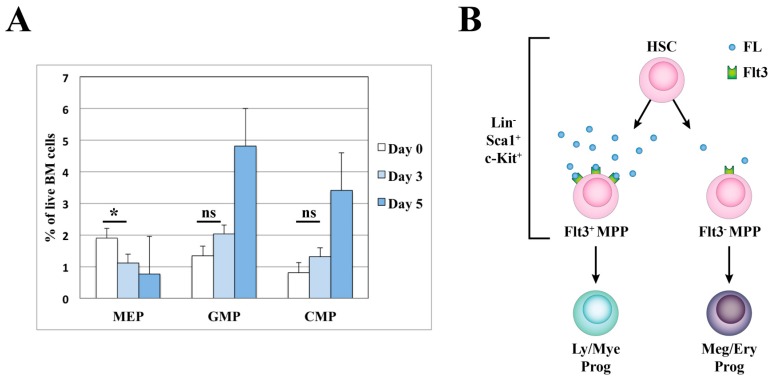
Potential instructive role of FL in early hematopoiesis. (**A**) Relative percentages of MEP, GMP and CMP populations in wild-type mice (*n* = 4) injected with 10 μg of FL daily, for a period of 5 days. Cells were pre-gated as Live, Lin^−^kit^+^Sca1/CD127^−^ and identified as: MEP: CD16^low^CD34^−^, GMP: CD16^+^CD34^+^, CMP: CD16^low^CD34^+^. *: significant (*p* = 0.017), ns: not significant. FL: Fms-like tyrosine kinase 3 ligand, MEP: Megakaryocyte-Erythrocyte Progenitor, GMP: Granulocyte-Macrophage Progenitor, CMP: Common Lymphoid Progenitor. BM: Bone Marrow. (**B**) Schematic representation of the proposed model for the instructive action of FL. HSC: Hematopoietic Stem Cell; MPP: Multipotent Progenitor; Ly/Mye Prog: Lymphoid/Myeloid Progenitor; Meg/Ery Prog: Megakaryocyte/Erythrocyte Progenitor; FL: Fms-like tyrosine kinase 3 ligand.

Prompted by the significant changes observed in mice repeatedly injected with FL [62,63], we have recently followed a reverse to the loss-of-function approach, by generating transgenic mice (hereafter FLtg mice) that express human FL under the control of the actin promoter, resulting in a sustained and high level expression of FL in vivo. As expected, FLtg mice exhibit a tremendous expansion of Flt3^+^ cells, including myeloid cells, DC, MPP, CMP, Granulocyte-Macrophage Progenitors (GMP), CLP and EPLM progenitors, resulting in leukocytosis in the blood and splenomegaly [64]. One of the most prominent phenotypes in these mice is a severe anemia that they develop quite early in life, due to reduced erythropoiesis, as demonstrated by the profound reduction in numbers of both Ter119^+^ enucleated erythroid progenitors as well as Megakaryocyte-Erythrocyte Progenitors (MEP) in the bone marrow. This negative effect of over-expression of FL on erythropoiesis could be due to an instructive action of FL, with the cytokine actively promoting the differentiation of early, multi-potent progenitors towards a lympho-myeloid fate. Alternatively, it could just be the result of a selective expansion of Flt3^+^ CMP and GMP progenitors at the expense of Flt3^−^ MEP, thereby leading to a growth disadvantage for megakaryocyte-erythrocyte progenitors and thus to their subsequent reduction. However, a kinetic analysis of these progenitors post injecting FL into wild-type mice showed that by day 3 Ter119^+^ erythrocyte progenitors are significantly reduced [64]. Considering the turnover of these progenitors and the fact that, at day 3 after FL injection, no other cell type shows any significant increase, this reduction in erythrocyte progenitors is more likely to be the result of a negative effect of FL on their generation, rather than a disadvantage in their expansion. Moreover, analysis of the MEP, CMP and GMP progenitors revealed that Flt3^−^ MEP are significantly reduced by day 3 of treatment and at a time when Flt3^+^ GMP and CMP are only slightly increased (Figure 2A). Even though absolute proof of an instructive or permissive action of a cytokine can only be derived from experiments at the single-cell level [65,66,67], careful analysis of our FLtg mouse model and of mice injected with FL provides strong in vivo evidence for an instructive role of FL in driving hematopoiesis towards the lympho-myeloid and away from the megakaryocyte/erythrocyte lineages. We suggest a working model for the role of FL in early hematopoiesis (Figure 2B) whereby activation of Flt3 signaling by FL above a certain level leads to up-regulation of lymphoid and myeloid lineage associated genes and a “priming” of progenitors to these cell fates. As a result of this priming Flt3 expression is further increased and the cells acquire the LMPP phenotype. In contrast, progenitors that do not receive a strong enough FL signal, either due to low Flt3 expression or due to low FL availability in their immediate microenvironment, will further down-regulate Flt3 and become “primed” to differentiate along the megakaryocyte/erythrocyte pathways, possibly under the influence of other cytokine signals, such as Erythropoietin (Epo) and Thrombopoietin (Tpo). This hypothesis could explain the discrepancy as to whether platelets and erythrocytes originate from Flt3^+^ progenitors or not, since it postulates that the level of Flt3 expression in vivo is a continuum and possibly under the influence of FL itself.

Interestingly, and considering the question of Flt3 expression on HSC, CD48^−^CD150^+^ HSC are significantly reduced in FLtg mice compared to wild-type [64]. However, it remains unknown whether this reduction is a direct effect of FL on HSC or a secondary effect due to aberrant expression of other cytokines in the FLtg bone marrow. In light of the recent data on Flt3 expression by HSC [54], identifying the precise stage where FL might exert its instructive action will be integral to elucidating the precise mechanism by which it does so.

### 3.2. Dendritic Cells

FL is essential to the generation of DC, as manifested by their severe reduction in the absence of active Flt3 signaling [35,68]. FL has a unique role as a factor that promotes the generation of both human and mouse DC, of all types, in bone marrow cultures [69,70,71,72,73]. Also, in vivo administration of FL in mice leads to a dramatic increase in numbers of both plasmacytoid and conventional DC [74,75,76]. The same effect is observed in transgenic mice expressing high levels of FL in either an inducible [77] or sustained manner [64,78]. The developmental origin of DC cell types is still not entirely resolved, but putative progenitor populations identified express the Flt3 receptor [79,80]. However, mature DC are also Flt3^+^ [70] and as a result it remains unclear whether FL regulates their generation from precursors, or expands them, or both.

Considering the central role of DC as antigen-presenting cells of the immune system, efforts have been made to utilize FL treatment in order to enhance immune response against malignancies. Accordingly, studies have shown a beneficial effect of FL administration against solid tumors [81,82,83], but the results from Phase I clinical trials have proved inconclusive [84,85]. Care should be taken in such therapeutic approaches, as FL would most probably have a growth-promoting effect on leukemic cells. In addition, FL-mediated DC expansion can be accompanied by increased proliferation of regulatory T cells (Treg) [63] and, therefore, a suppression of the desired anti-tumor immune response.

### 3.3. B Cells

As discussed before, one of the phenotypes of both FL^−/−^ and *Flt3*^−/−^ mice is a significant reduction in the numbers of B cell progenitors in the bone marrow, while early in vitro experiments showed a positive effect of FL in B cell generation. Furthermore, FL enhances reconstitution of the B cell compartment after irradiation or chemically-induced myeloablation [86] and appears to be a critical factor for foetal B lymphopoiesis as well [87]. It has been shown that the B cell commitment transcription factor Pax5, which is responsible for the irreversible commitment of pro-B cells to the B cell lineage [88,89], downregulates Flt3 expression [90]. Therefore, FL cannot promote the expansion of committed B cell progenitors that express CD19 (another Pax5 target) but its positive effect on B cell generation should rather be attributed to a role in early, un-committed precursors that generate the B cell pool. Indeed, absence of FL signaling leads to a decrease in the numbers of CLP and EPLM progenitors [36,37,38,39], the Ly6D^+^ fraction of which is Flt3^+^ and represents the latest un-committed B cell progenitors prior to Pax5 expression [91,92].

FL has been shown to promote the survival of Flt3^+^CD19^−^ progenitors, as over-expressing the pro-survival gene *Bcl2* in FL^−/−^ mice can significantly restore their numbers [37,39]. However, this rescue is only partial and even though numbers are increased, B cell priming is not restored in these progenitors. It should be noted that due to the anti-proliferative effect of Bcl2 [37,93] the potential role of FL as a survival factor for Flt3^+^CD19^−^ progenitors might not have been fully assessed in these studies. A more clear role of FL in promoting the proliferation of these progenitors has been revealed, as FL over-expression increased the percentage of cycling Ly6D^+^ EPLM, while FL deficiency had exactly the opposite effect [37]. Therefore, FL seems to be pivotal for promoting the expansion and possibly the survival of Flt3^+^CD19^−^ un-committed progenitors, which will further differentiate to committed B cell progenitors. But does FL also act as an instructive cytokine for this commitment? The findings that neither Bcl2 over-expression in FL^−/−^ mice [39], nor increased availability of FL itself [37] lead to increased B cell priming in early CLP/EPLM progenitors argue against such an instructive role. On the contrary, FLtg mice exhibit a reduction in the percentage of B-lineage skewed Ly6D^+^ EPLM cells expressing Pax5 [37]. Thus, FL seems to exert only a permissive role in B cell development, by promoting the proliferation and survival of early progenitors, therefore facilitating the generation of a significant number of CLP/EPLM precursors, some of which will eventually commit to the B cell fate through Pax5 up-regulation. Pax5 will in turn shut down Flt3 expression in CD19^+^ committed pro-B cells. Considering the above discussed evidence for an instructive role of FL in early hematopoietic lineage decisions, this permissive role in B cell development highlights the functional versatility of hematopoietic cytokines, since their action can be very much cell context dependent.

### 3.4. T Cells

Thymopoiesis seems to be largely unaffected in FL^−/−^ or *Flt3*^−/−^ mice. However, and in keeping with the notion of the synergistic action of FL with other cytokines, ablation of Flt3 signaling further exacerbates the defect in T cell development observed in Interleukin-7 (IL-7) knock-out mice [94]. This is probably due to the fact that the bone marrow progenitors that seed the thymus are Flt3^+^ and the earliest identified thymic T cell progenitors also express Flt3 [95]. Indeed, FL treatment of immunodeficient mice after bone marrow transplantation accelerates T cell recovery because it expands bone marrow Flt3^+^ LSK progenitors [96]. Further, this Flt3 expression by the thymus seeding progenitors seems to be functionally important, since FL production by the thymic microenvironment has been shown to promote the maintenance of these progenitors, both in steady-state conditions and after irradiation [97,98]. Interestingly, high levels of FL in vivo lead to an increase in the numbers of Treg [63,64], but this seems to be an indirect effect, through an IL-2 dependent activation of Treg proliferation following interactions with DC, a cell type that is vastly increased under conditions of increased FL availability [63].

### 3.5. Overview

Overall, our current knowledge points towards a critical role of FL in the generation of lymphoid cells. However, this role is mainly exerted on early, un-committed lymphoid progenitors and it seems to be of a permissive nature, i.e., promoting their proliferation and survival. Commitment and further maturation of lymphoid cells coincides with downregulation of Flt3, thereby rendering them unresponsive to FL. Apart from B and T cells, this seems to hold true for Innate Lymphoid cells (ILC) as well, since FL regulates their numbers through the maintenance of their early progenitors [99,100]. Even though the intracellular response to FL might be different for different lymphoid lineages, this common pattern of Flt3 downregulation upon commitment and maturation to functional lymphocytes seems to indicate that active Flt3 signaling possibly contributes to the maintenance of an “immature” lymphoid phenotype and therefore it needs to be silenced in order for mature lymphoid cells to become functional. There have been reports of Flt3 re-expression on mature, activated B cells [101,102] and activated human T cells [103]. For B cells, this could be the result of Pax5 downregulation, which is necessary for further differentiation to antibody secreting plasma cells. Some evidence for a potential functional significance of Flt3 expression on activated lymphocytes has been reported [102] but further investigations are required to elucidate the exact role of FL in this context.

## 4. FL and Flt3 in Hematopoietic Malignancies

Aberrant expression of FLT3 is very commonly found in hematopoietic malignancies. In most cases, this is due to activating mutations in the *FLT3* gene but a significant number of leukemias are also characterized by a higher than normal expression level of un-mutated, wild-type FLT3, thus underscoring the importance of FLT3 signaling perturbations in malignant transformation. In most cases FLT3 mutations are associated with a poor clinical prognosis [104]. FLT3 mutations are most commonly found in AML patients, almost one third of which harbor such a mutation. In addition, 5–10% of patients with Myelodysplasia (MDS) and 1–3% of Acute Lymphoblastic Leukemia (ALL) patients have mutations in the *FLT3* gene. In pediatric leukemias FLT3 mutations are somewhat more rare, but they also clearly associate with poor clinical prognosis [105].

There are two types of FLT3 mutations found in AML: internal tandem duplications of the juxta-membrane domain (the mutated receptor thus termed FLT3-ITD) and point mutations in the tyrosine kinase domains (collectively named FLT3-TKD).

### 4.1. FLT3-ITD

**Figure 3:**
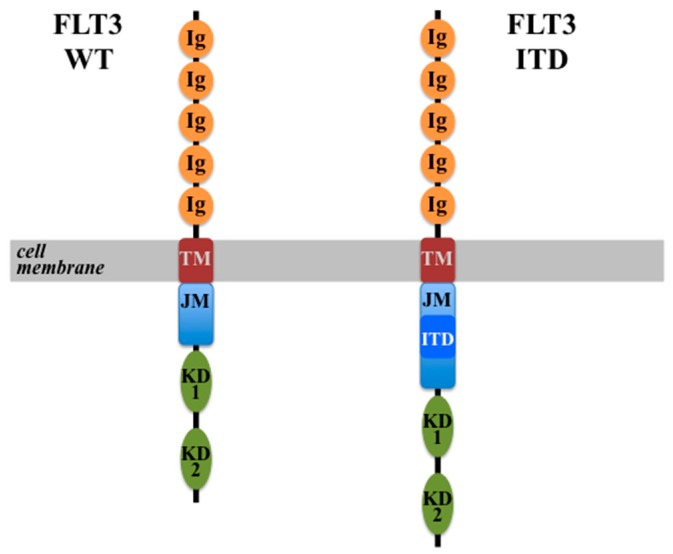
Schematic diagram of the structure of wild-type FLT3 (**left**) and FLT3-ITD (**right**) receptors. Ig: Immunoglobulin-like domain; TM: transmembrane domain; JM: juxta-membrane domain; KD: kinase domain; ITD: internal tandem duplications.

In 1996, Nakao et al. described for the first time the presence of a mutated FLT3 receptor in AML patients, which exhibited tandem duplications in the juxta-membrane domain [106]. Since then this particular type of mutation has been studied extensively and identified as one of the most common mutations in AML [107,108,109]. The mutation consists of a head-to-tail replication of sequences coding part of the juxta-membrane domain of the receptor (Figure 3). These sequences can be variable in length (from 3 to >400 base pairs) and, as they are always found to be in-frame, they result in the transcription and translation of a receptor with an elongated juxta-membrane domain. The consequence of this elongation is that the mutated receptor can dimerize, phosphorylate and activate the kinase domains without the need to bind FL, therefore resulting in ligand-independent, constitutive activation of FLT3 [110,111,112]. This probably occurs by eliminating an auto-inhibitory function of the wild-type receptor, which ensures that, without ligand binding and dimerization, the kinase domains cannot be activated [110,113]. It has been proposed that these tandem duplications occur as a result of DNA replication mistakes and they provide a growth advantage to the cells harboring them [111].

Studies on the signaling events occurring downstream of the constitutively activated FLT3-ITD have initially been carried out using cell lines transduced with the mutated receptor and have indicated that there are qualitative differences between FLT3-ITD and wild-type FLT3 signaling. In addition to activating the Ras/MEK/Erk and PI3K pathways [111,112], which are also activated by wild-type FLT3, FLT3-ITD has been shown to promote STAT5 phosphorylation and subsequent DNA binding [114]. As a consequence, FLT3-ITD can activate an array of STAT5 target genes, which would normally not be expressed upon binding of FL to its wild-type receptor [115]. Interestingly, amongst them are not only cell-cycle regulating genes, which would account for a growth advantage of FLT3-ITD harboring cells, but also myeloid differentiation transcription factors, such as PU1 and C/EBPα, which seem to be suppressed by FLT3-ITD [115,116]. Another outcome of the aberrant STAT5 activation triggered by FLT3-ITD seems to be an increase in reactive oxygen species production and in the frequency of double-strand DNA breaks, therefore resulting in genomic instability and providing a potential mechanism for the apparent poor clinical prognosis of patients harboring FLT3-ITD mutations [117,118]. In addition, a potential mechanism by which FLT3-ITD might promote survival and proliferation of AML cells is through phosphorylation and subsequent suppression of the Forkhead family of transcription factors member FOXO3a, an important pro-apoptotic regulator [119].

Important insights into the mechanisms by which FLT3-ITD can promote leukemogenesis have come from in vivo studies. Apart from injecting FLT3-ITD transduced cell lines into mice [114,120], early attempts to create mouse models where the mechanism of FLT3-ITD leukemogenesis could be studied involved retroviral transduction of bone marrow cells and subsequent transplantation into recipient mice. These experiments showed that FLT3-ITD expressing bone marrow cells caused a myeloproliferative disease in the recipient mice, characterized by splenomegaly, leukocytosis and expansion of myeloid lineages, but without developing an AML phenotype similar to human patients [121,122]. Similar results were obtained from a transgenic mouse model expressing FLT3-ITD under the control of the *vav* promoter, with the difference that few transgenic lines in this system developed a lymphoid disease as well [123]. Mouse models that resemble more closely the leukemogenic effect of FLT3-ITD in humans have been generated by two groups, through a knock-in approach, whereby the human FLT3-ITD gene was inserted into the endogenous *Flt3* locus [124,125]. In both studies, mice developed symptoms of myeloproliferative disease, with splenomegaly, leukocytosis, expansion of myeloid progenitors and dendritic cells, as well as a decrease in the numbers of B cell progenitors. Interestingly, while both mouse models exhibited a significant increase in early LSK progenitors, further analysis of LSK subpopulations in one of them revealed a reduction in the number of CD48^−^CD150^+^ HSC [126]. This reduction was shown to be the result of increased proliferation and cell-cycle entry of HSC, eventually leading to their exhaustion. Considering that FLT3-ITD in this mouse model is expressed under the control of the endogenous *Flt*3 regulatory elements, these results would argue in favor of Flt3 expression within the HSC compartment, as discussed previously (Figure 1) [54].

### 4.2. FLT3-TKD

Point mutations in the tyrosine kinase domain of FLT3 are the second most common type of FLT3 mutations found in AML. They have also been associated with an unfavorable clinical outcome in patients although, due to their somewhat lower frequency, very large studies are needed to precisely evaluate their impact on clinical outcome [113]. FLT3-TKD point mutations also promote ligand-independent phosphorylation of the receptor and cell growth [127,128]. In wild-type FLT3, in the absence of ligand binding, the activation loop of the kinase domain remains in a closed conformation, therefore preventing ATP and protein binding [129]. It is believed that TKD mutations result in opening of this region and subsequent activation without the need for ligand binding. Even though both FLT3-ITD and FLT3-TKD mutations confer ligand-independent receptor activation, the mechanisms by which they contribute to the development of leukemia might be different. Indeed, signaling downstream of both mutated receptors seems to differ as to which signal transduction pathways get activated [130], and a study comparing gene expression profiles of the two types of mutations in childhood AML patients showed significant differences in the genetic program they induce [131]. FLT3-ITD has been shown to localize to a large extent intra-cellularly [132], resulting in aberrant interactions with signaling molecules [133], and this has been hypothesized to be one mechanism responsible for differential signaling by FLT3-ITD and FLT3-TKD [134]. Furthermore, in a mouse bone marrow transplantation model, FLT3-TKD not only manifested a malignancy with longer latency compared to FLT3-ITD, but it was also found to cause an oligoclonal lymphoid disease, in contrast to FLT3-ITD, which led to the development of a myeloproliferative disorder [121]. The reason for the difference in lineage outcome of the disease was differential activation of STAT5 from the two types of mutations, since the FLT3-TKD mutation expressed in this model did not result in STAT5 phosphorylation, as was the case with FLT3-ITD. Intriguingly, STAT5 phosphorylation seemed to be the decisive factor as to the lineage phenotype of the disorder, since deletion of STAT5 in FLT3-ITD-mediated malignancy significantly increased survival and switched the immunophenotype of the disease from a myeloid to a lymphoid one [135]. The differences in the in vivo effects of FLT3-ITD and FLT3-TKD were also demonstrated in a knock-in mouse model expressing the FLT3-TKD most commonly found in AML [136]. In agreement with the previous study, this mouse model showed that FLT3-TKD manifested a less aggressive malignancy than FLT3-ITD, and even though myeloid progenitors were increased, a significant expansion of B cell progenitors was also observed.

Apart from activating mutations, increased expression levels of the wild-type FLT3 receptor have also been observed in cases of leukemia [137,138,139,140]. Consequently, high FLT3 expression and/or constitutively active mutated FLT3 are found in 70–100% of AML cases, as well as in a high percentage of ALL cases [104]. The importance or potential mechanism of high FLT3 expression in leukemias remains unknown. Some receptor tyrosine kinases can exhibit dimerization even in the absence of ligand binding [141]. Therefore, a significant increase in the amount of FLT3 on the cell surface might facilitate some degree of ligand-independent dimerization and activation of the receptor [142]. Alternatively, it could be hypothesized that in steady-state conditions the amount of FLT3 on the cell surface is the limiting factor for FLT3 signaling, with FL being abundant in the bone marrow. Therefore, higher FLT3 expression could result in stronger downstream signaling. In that context, it should be noted that there is evidence for autocrine FL signaling from studies of AML patients’ cells and leukemic cell lines [143].

### 4.3. FLT3 Inhibitors

The poor response of FLT3-ITD AML patients to conventional therapies has prompted investigation of the use of FLT3 tyrosine kinase inhibitors (TKIs) to treat relapsed and refractory AML. Interestingly, blast cells from FLT3-ITD AML patients at the time of relapse are more sensitive to FLT3 TKIs when compared to presentation blasts [144]. This suggests that chemotherapy selects for cells that are dependent on FLT3-ITD signalling, underscoring the potential of FLT3 TKIs in treating relapsed AML.

First generation FLT3 inhibitors were non-specific TKIs and inhibited other tyrosine kinase receptors, such as c-KIT and vascular endothelial growth factor receptor [145,146,147,148]. These include two TKIs, sunitinib and sorfenib, which have been approved for the treatment of solid tumours [149,150,151,152,153]. Phase I and Phase I/II clinical trials have demonstrated that sunitinib inhibits FLT3 signalling in AML patients’ cells [154] and is well tolerated as both a monotherapy and with intensive chemotherapy [155,156]. As a monotherapy, sunitinib induces short-term (4–16 weeks) and partial remission [156]. Sunitinib with conventional chemotherapy seems promising, but this combination has only been investigated in a single-arm Phase I/II study [155]. Sorefenib has been more intensely studied as to its use in AML. Early studies of sorefenib as a monotherapy to treat AML generated conflicting outcomes [157,158,159]. However, when combined with chemotherapy, sorefenib has been shown to increase event- and relapse-free survival in patients with previously untreated AML who are above, but not below, the age of 60 [160], though toxicity is increased [161,162]. Sorafenib also sustains remission in AML patients harbouring FLT3-ITD mutations following allogenic stem cell transplant [163,164] and a Phase IV trial is currently underway to investigate this further (NCT02474290). Midostaurin is another FLT3 TKI that has shown promise in young AML patients. Stone et al. have shown that midostaurin with intense chemotherapy increases both event-free and overall survival in patients with newly diagnosed AML (≤60 years) [165,166]. Lestaurnib has been studied as a potential treatment for AML but has shown limited clinical benefit and achieving sustained FLT3 inhibition with lestaurnib has proven challenging [167,168]. Second generation FLT3 TKIs are highly selective for FLT3 and have shown significant promise in treating relapsed and refractory AML. Quizartnib is one of the most effective monotherapies for the treatment of FLT3-ITD AML and in one Phase II study complete remission was seen in approximately half of the patients and was sustained for an average of 11–13 weeks [169,170]. A pilot study has also shown that quizartnib is an effective treatment of newly diagnosed AML when combined with chemotherapy (a complete remission seen in 79% of patients) [171], and a phase III clinical trial is currently investigating the use of quizartnib in combination with chemotherapy in newly diagnosed FLT3-ITD AML (NCT02668653). Two other second generation FLT3 TKIs, gilteritinib and crenolanib, have also been shown to have activity against AML cells with FLT3-ITD and FLT3-TKD mutations. A phase I/II clinical trial has demonstrated that gilteritinib is effective as a monotherapy in the treatment of relapsed and refractory AML [172]. Complete remission was observed in 49% and 29% of FLT3-ITD and FLT3-TKD AML patients, respectively. Crenolanib is effective in treating relapsed and refractory AML [173] and has been shown to be particularly useful in the treatment of cases of AML that are resistant to previous TKI therapy [174,175,176]. In a recent trial of crenolanib in patients with relapsed or refractory AML harbouring *FLT3* mutations, the overall response rate was 31% in patients that had received prior FLT3 TKI treatment and 39% in FLT3 TKI naïve patients [177].

Mechanisms of FLT3-TKI resistance in AML have been identified, such as the FLT3-TKD mutations that commonly confer resistance to quizartnib [178,179]. One proposed mechanism is that elevated plasma FL levels that are induced following chemotherapy reduce the activity of FLT3-TKIs, and this is supported by in vitro studies [180]. Therefore, several studies have investigated the use of FLT3-TKIs with azacytidine, a hypomethylating agent that reduces plasma FL concentrations, in the treatment of AML. An interim report of a study examining the combination of quizartinib with azacitidine has shown promising results, with a response observed in 69% of patients with relapsed or refractory MDS, chronic myelomonocytic leukemia or AML, including 4 of 7 patients that had received prior FLT3 TKI treatment [181]. Another approach to overcoming resistance is the use of antibodies to FL in combination with FLT3-TKIs ([182] and NCT02789254).

## 5. Open Questions and Future Challenges

The large amount of data briefly discussed above have significantly increased our knowledge as to the role of the FL-FLT3 axis, both in normal and malignant hematopoeisis. However, several questions with clinical importance remain open. It is clear that activating mutations of FLT3 are an important factor for disease progression in AML. Nevertheless, mouse models indicate that the presence of a FLT3 mutation alone might not be sufficient to cause leukemia. It has been hypothesized, in accordance with the “two hit” model for neoplastic transformation [183], that FLT3 mutations have to act in concert with at least one other mutation, in order for hematopoietic cells to become leukemic. This hypothesis postulates that aberrant FLT3 signaling would provide the increased survival and growth signal to cells, while the additional mutation would confer a differentiation block, therefore resulting in the transformation of the cell to a leukemic blast. In support of this hypothesis, it has been shown that FLT3-ITD can cause a leukemia-like phenotype in a mouse model only when present in combination with the t(15;17) translocation [184] and FLT3-ITD is frequently found in leukemias together with genetic rearrangements and point mutations [104].

Several lines of evidence point towards a very important role of FL in lymphoid development. Absence of Flt3 signaling seems to affect lymphoid cells more than myeloid [34,35], while high expression of Flt3 in MPP has identified a progenitor population with more robust differentiation potential for lymphoid rather than myeloid cells [48]. However, FLT3 mutations are found mainly in myeloid leukemias and mouse models have shown that such mutations predominantly confer a myeloproliferative rather than lymphoproliferative disease. This remains a paradox, though it can be hypothesized that the key to answering this question lies in the specific signaling pathways and genes that the mutated receptor activates in comparison to the wild-type one. Therefore, further investigations are needed in this direction—not only at the gene expression level, since a lot of important differences between wild-type and FLT3-ITD might occur at a post-translational level. Recent evidence would suggest that the activation of STAT5 specifically by FLT3-ITD might be very important in this context [135]. Further research into the mechanistic aspects of this lineage-specific leukemogenic transformation by mutated FLT3 is required, with the final aim to assist the development of specific inhibitors for therapeutic treatment.

It is interesting that the phenotype caused by expression of mutated FLT3 in mouse models highly resembles the one observed in mice with high levels of FL; i.e., leukocytosis, splenomegaly, anemia, expansion of DC and myeloid progenitors and reduced B cell generation [64,78]. It is tempting to hypothesize that constitutive, ligand-independent Flt3 signaling might elicit a cellular response that is similar to the one caused by activation of the receptor due to sustained FL exposure. To this end, it will be interesting to assess whether continuous wild-type Flt3 engagement might trigger downstream signals that are significantly different from the ones generated by transient or low-level signaling. This could be of particular interest for potential therapeutic applications in leukemias with wild-type FLT3 over-expression.

Further investigation of the role of FL in normal hematopoiesis might provide clues as to the manner in which FLT3-related leukemias can be treated. However, the reverse is also true, since studies on mutated FLT3 biology can provide insights on the physiological role of the cytokine. A recent study showed that FLT3-ITD can instruct myeloid differentiation in lympho-myeloid oligo-potent progenitors in a mouse model [185]. Apart from providing further evidence and a potential mechanism for the presence of FLT3-ITD mainly in myeloid leukemias, this study also provides some support for the hypothesis of an instructive role of growth factor receptor signaling in early hematopoiesis. Another example is the finding that FLT3-ITD expressed under the control of the endogenous *Flt3* promoter directly affects HSC proliferation in vivo, thus supporting the notion that Flt3 expression is initiated already in HSC (Figure 1).

The studies that have been briefly outlined herein have greatly advanced our understanding not only of how the cytokine FL exerts its function in hematopoiesis, but also of how normal blood cell generation is regulated. The clinical importance of Flt3 signaling requires further investigations into the role of FL and its receptor in leukemia.
